# Arthroscopic reduction of an irreducible distal radioulnar joint in Galeazzi fracture-dislocation due to a fragment of the ulnar styloid: a case report

**DOI:** 10.1186/s12891-019-2735-5

**Published:** 2019-07-31

**Authors:** Masayoshi Iwamae, Koichi Yano, Yasunori Kaneshiro, Hideki Sakanaka

**Affiliations:** Department of Orthopaedic Surgery, Seikeikai Hospital, 1-1-1 Minamiyasuicho, Sakai-ku, Sakai City, Osaka, 590-0064 Japan

**Keywords:** Galeazzi fracture-dislocation, Distal radioulnar joint, Arthroscopy, Minimal invasion, Irreducible

## Abstract

**Background:**

There are only a few published case reports of irreducible Galeazzi fracture-dislocation, and patients in these studies had undergone reduction by open surgical methods. Arthroscopy for the distal radioulnar joint of the wrist joint has recently been used for wrist pathology. We aim to describe the surgical procedure involved in arthroscopic reduction of irreducible Galeazzi fracture-dislocation and clinical outcome and review the literature.

**Case presentation:**

We present the case of a 26-year-old man, a professional athlete, who sustained Galeazzi fracture-dislocation during a bicycle race. The distal radioulnar joint was irreducible because the fragment of the ulnar styloid was trapped between the sigmoid notch and ulnar head after a doctor had previously reduced it manually. Operative treatment was performed using a 30° oblique, 1.9-mm arthroscope. Reduction of the fragment of the ulnar styloid was achieved using distal radioulnar joint arthroscopy. The metaphyseal and intra-articular fracture of the radius and the fragment of the ulnar styloid were fixed using a volar locking plate and tension band wiring technique, respectively. A daily injection of parathyroid hormone and low-intensity pulsed ultrasound were used postoperatively. The patient was asymptomatic and returned to the preinjury level of athletic activity 2 months postoperatively, and bone union of the radius and ulna was achieved without distal radioulnar joint instability 15 months postoperatively.

**Conclusions:**

Less invasive reduction of the dorsal anatomical structure enabled our patient to return early to sports. We consider arthroscopic reduction to be superior to the open surgical method in terms of evaluating interpositions; additionally, arthroscopic reduction is minimally invasive and does not need immobilization because it does not cause significant damage to the dorsal capsule and subsheath of the extensor carpi ulnaris, which comprise the triangular fibrocartilage complex.

## Background

There are only a few published case reports of irreducible Galeazzi fracture-dislocation, and patients in these studies had undergone reduction by open surgical methods [1–9]. Herein, we present a rare case of an irreducible distal radioulnar joint (DRUJ) due to entrapment of a fragment of the ulnar styloid between the sigmoid notch and ulnar head. The fragment of the ulnar styloid was reduced successfully by arthroscopy through the DRUJ, and this procedure was less invasive. The purposes of this case report are to describe the surgical procedures and clinical outcome of our patient, and to review the literature.

## Case presentation

**Fig. 1:**
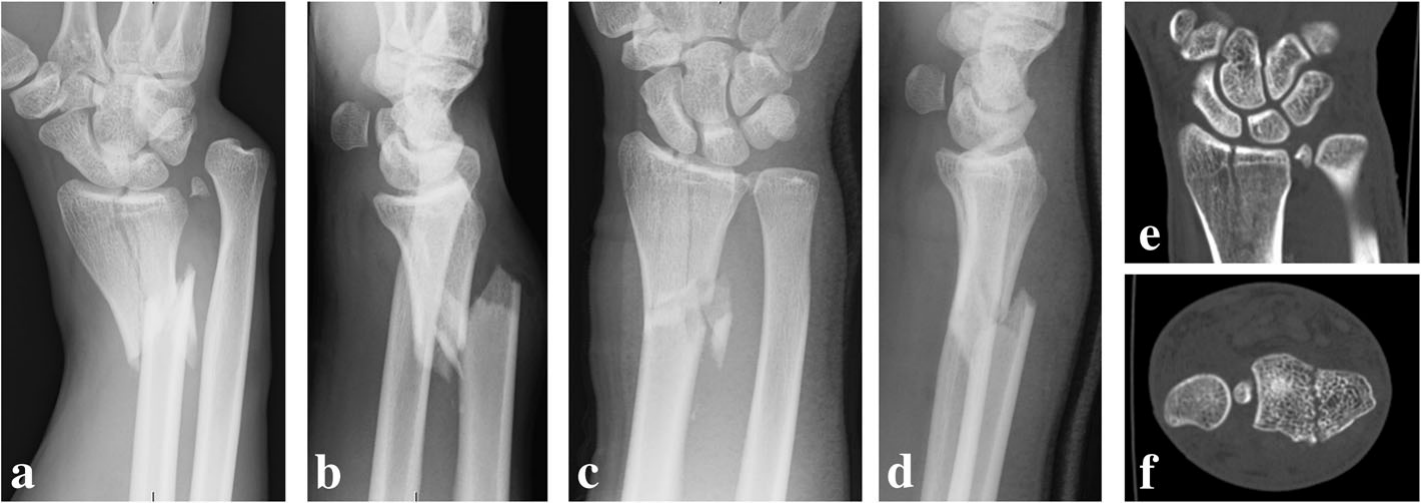
Plain radiograph and plain computed tomography (CT) scan of the right wrist before and after manual reduction. (**a**) Anteroposterior (AP) view and (**b**) lateral view before manual reduction. (**c**) AP and (**d**) lateral views after manual reduction. (**e**) Coronal view and (**f**) axial view of the CT image of the distal radioulnar joint. The fragment of the ulnar styloid is trapped between the sigmoid notch and ulnar head

Informed consent for publication was obtained from the patient, and this case report was approved by the institutional review board. A 26-year-old right-handed man sustained right wrist pain during a professional bicycle race. He had no remarkable medical history. A doctor he met had diagnosed him as having Galeazzi fracture-dislocation, and it was reduced manually (Fig. 1a, b). Subsequently, he was referred to us for further treatment. Preoperative plain radiography and computed tomography of the right wrist joint showed that the metaphyseal and articular fracture of the radius and the fragment of the ulnar styloid were trapped between the sigmoid notch and ulnar head (Fig. 1c-f).

**Fig. 2:**
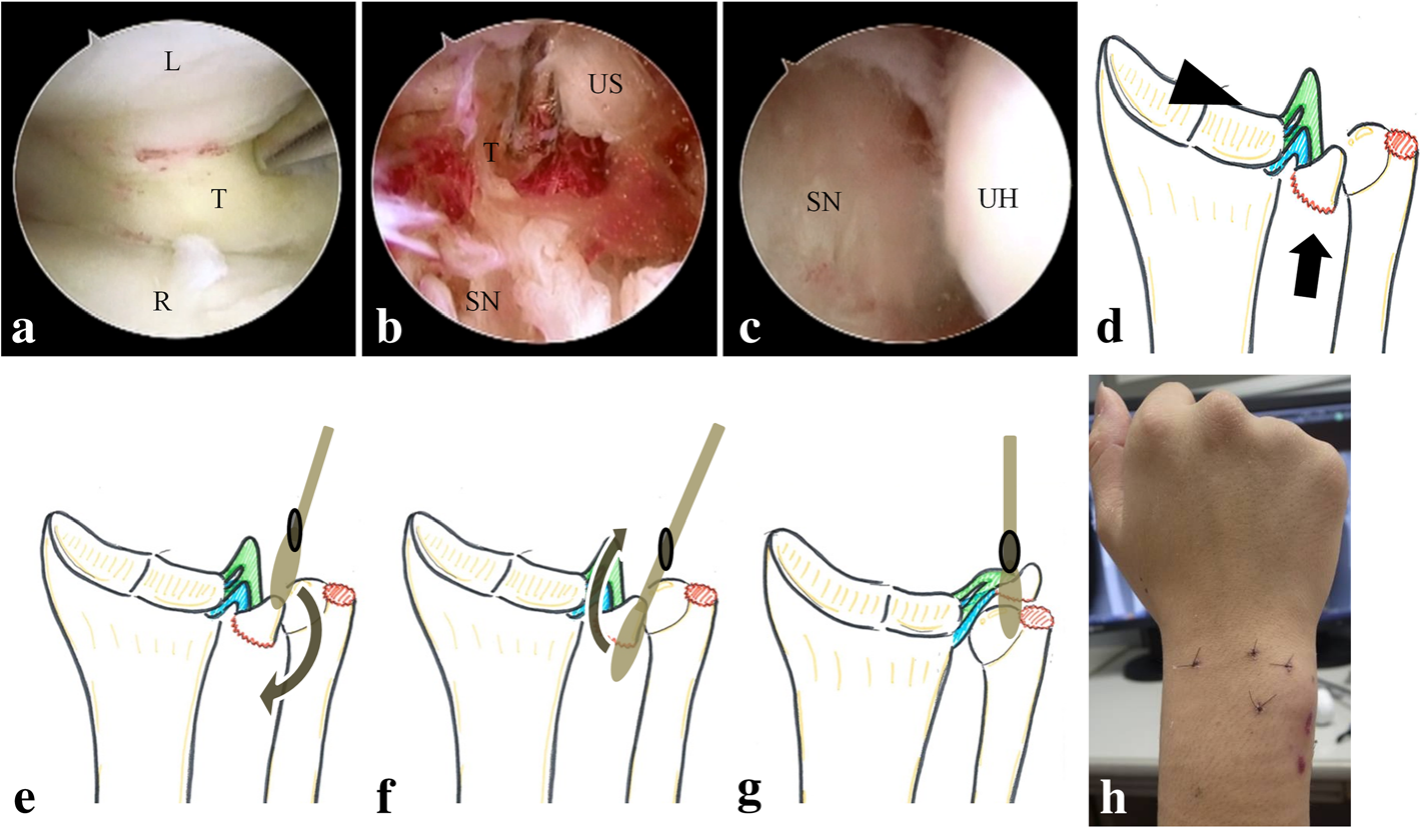
Photographs and schema of the wrist during and after arthroscopic treatment. (**a**) Arthroscopic view from the 3–4 portal. The triangular fibrocartilage complex (TFCC) extends over to the radial side. (**b**) Arthroscopic view from the proximal distal radioulnar joint (DRUJ) portal. A fragment of the ulnar styloid is trapped between the sigmoid notch and ulnar head. The TFCC extends to the fragment. (**c**) Arthroscopic view from the proximal DRUJ portal. The sigmoid notch faces the ulnar head after the ulnar styloid is reduced. (**d**) Schema of the arthroscopic procedure from (**a**) to (**c**). The arrowhead indicates the view from the 3–4 portal (**a**), and the arrow indicates the view from the proximal DRUJ portal (**b**) and (**c**). (**e**-**g**) Schema of the procedures of reduction. (**e**) The elevatrium from the distal DRUJ portal was brought under the entrapped fragment of the ulnar styloid by guiding it to the radial side of the ulnar head. (**f**) The fragment of the ulnar styloid was lifted up by the elevatrium. (**g**) The entrapment of the fragment of the ulnar styloid was reduced. (**h**) Postoperative site of the dorsal wrist. L, lunate; T, triangular fibrocartilage complex; R, radius; US, ulnar styloid; SN, sigmoid notch; UH, ulnar head

**Fig. 3:**
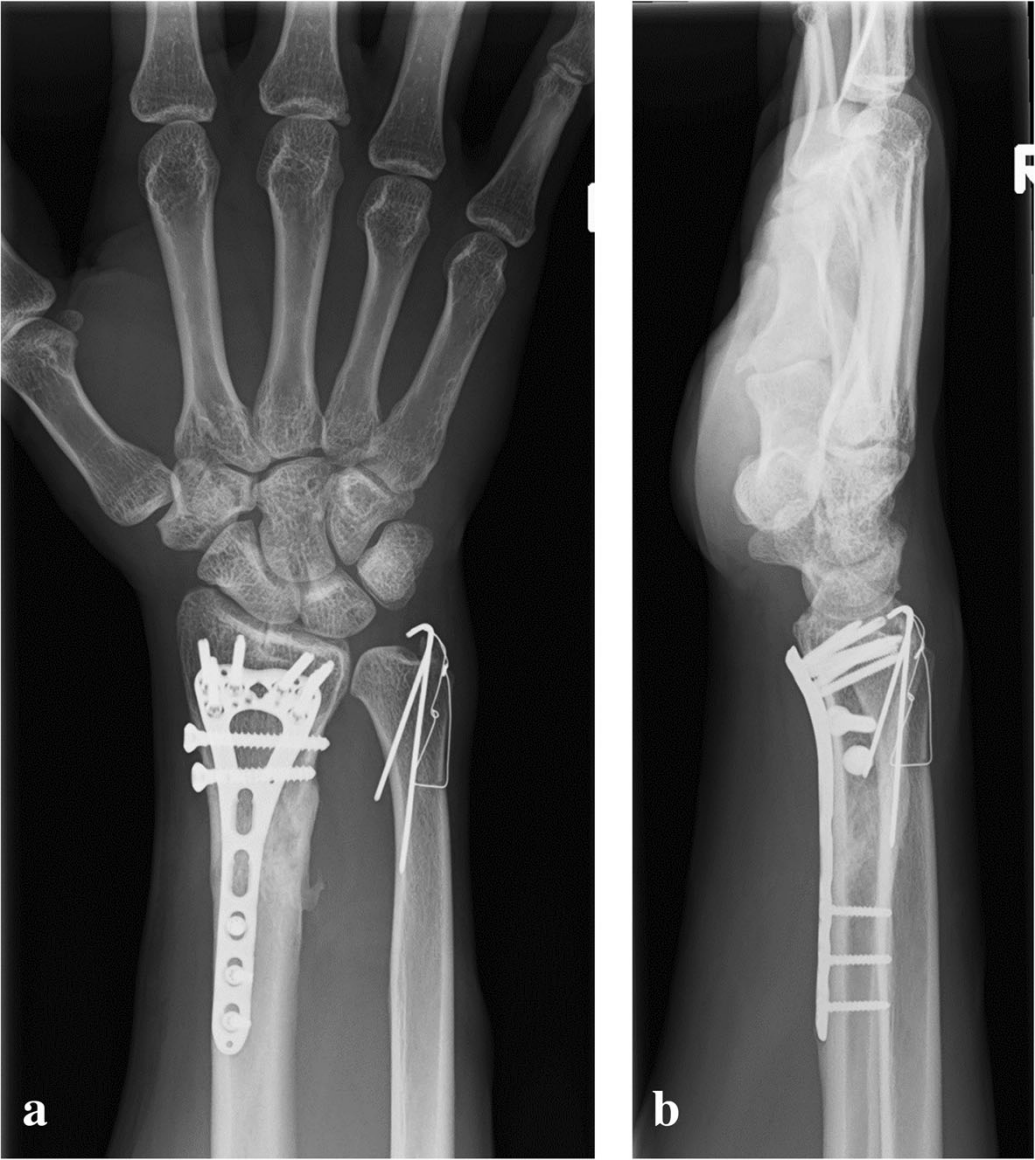
Plain radiograph of the right wrist at 6 months postoperatively. (**a**) Anteroposterior view and (**b**) lateral view. Bone union of the radius and ulna is achieved

Operative treatment was performed under general anesthesia with air tourniquet control. After the radius was exposed by the trans-flexor carpi radialis approach and reduced with a bone clamp, we evaluated the reduction of the articular surface of the radius by arthroscopy using 3–4 and 4–5 portals in the radiocarpal joint using a 30° oblique, 1.9-mm arthroscope (Stryker K.K., Tokyo, Japan). There were less than a 1-mm gap and step-off in the articular surface; therefore, we judged that the articular surface was acceptably reduced. We tried to reduce the fragment of the ulnar styloid, but we could not visualize the ulnar side because of the triangular fibrocartilage complex (TFCC), which extended to the radial side (Fig. 2a). Next, arthroscopy of the DRUJ was performed using distal and proximal DRUJ portals, and this showed ligamentous tissue, the TFCC, the sigmoid notch, and the cancellous bone, which was supposed to be the base of the ulnar styloid fragment (Fig. 2b). Using the viewing portal from the proximal DRUJ portal, we inserted an elevatrium from the distal DRUJ portal. The elevatrium was brought under the fragment of the ulnar styloid by guiding it to the radial side of the ulnar head, and the fragment of the ulnar styloid was successfully reduced by lifting up the elevatrium. (Fig. 2e-g) After reducing the fragment, the sigmoid notch faced the ulnar head (Fig. 2c). Subsequently, the metaphyseal and articular fractures, including the third fragment of the radius, were fixed with a volar locking plate (2.4-mm Variable Angle LCP Two-Column Volar Distal Radius Plate; DePuy Synthes, Tokyo, Japan). Next, the distal ulna was approached from the ulnar side of the wrist, and the fragment of the ulnar styloid containing the distal and proximal components of the TFCC was avulsed from the fovea of the ulna and fixed using the tension band wiring (TBW) technique (Fig. 3).

**Fig. 4:**
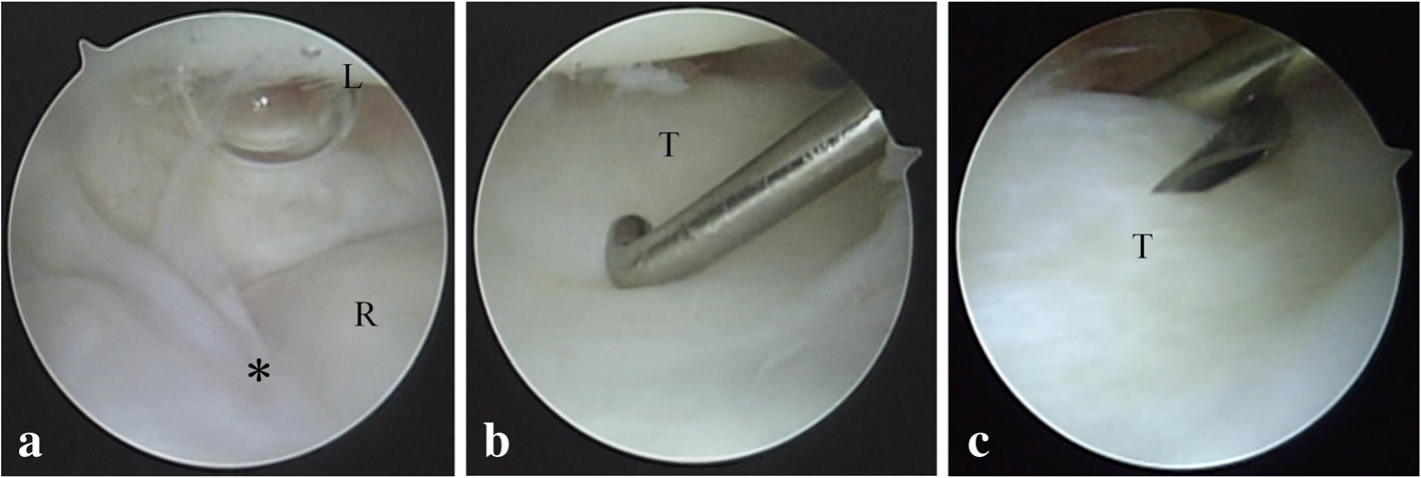
Intraoperative photograph of arthroscopy at the second operation. (**a**) Articular surface of the intra-articular fracture of the radius. Asterisk shows a primary fracture line. (**b**) The trampoline test and (**c**) hook test for the assessment of the stability of the triangular fibrocartilage complex (TFCC). TFCC using a probe. L, lunate; T, triangular fibrocartilage complex; R, radius

**Fig. 5:**
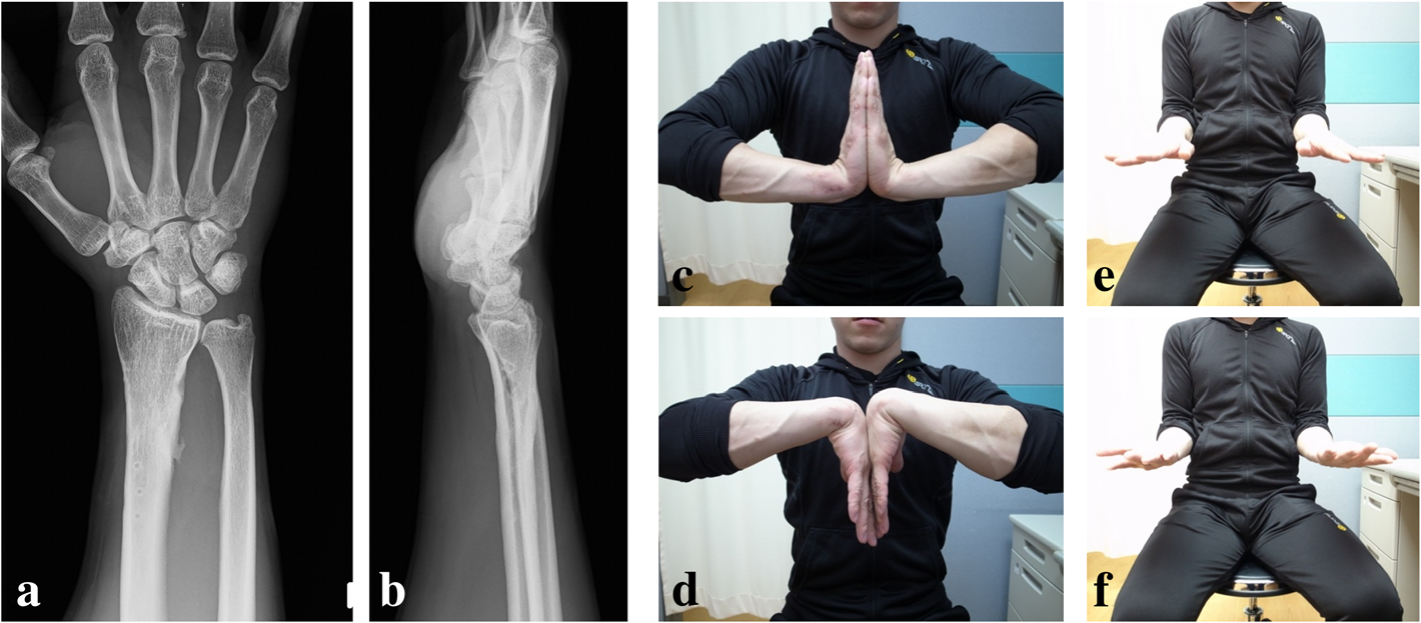
Plain radiograph and clinical photograph at the final follow-up 15 months postoperatively. (**a**) Anteroposterior view and (**b**) lateral view of the right wrist. (**c**) Dorsal flexion of the wrist, (**d**) palmar flexion of the wrist, (**e**) pronation of the forearm, and (**f**) supination of the forearm

The patient’s postoperative course was uneventful. We applied a bulky dressing for 2 days, and exercises of the finger, wrist, and forearm were started the day after surgery. A daily injection of parathyroid hormone (PTH) (20 μg of teriparatide; Eli Lilly Japan K.K., Kobe, Japan) was administered 2 days postoperatively, and low-intensity pulsed ultrasound (LIPUS) (SAFHS 4000 J; Teijin Pharma Ltd., Tokyo, Japan) was performed 4 days postoperatively because he wanted an early return to competitive bicycle racing. He returned to professional bicycle racing 2 months postoperatively. Three months postoperatively, bone union was achieved. Because he complained of discomfort of the ulnar wrist due to irritation from the TBW, we performed implant removal of the radius and ulna and arthroscopy of the radiocarpal joint 9 months postoperatively. The arthroscopic findings showed that the TFCC maintained normal tension, as judged by results of a hook test, and the articular surface of the radius was repaired without cartilage injury [10]. (Fig. 4) At the final follow-up 15 months postoperatively, the patient was asymptomatic and returned to the preinjury level of athletic activity. Plain radiography showed bone union of the radius and ulna without osteoarthritic changes (Fig. 5a, b). Physical examination showed that the DRUJ was stable, and the patient’s grip strengths were 64.0 kg and 60.6 kg for the right and left hands, respectively. The range of motion of the right and left extremities were as follows, respectively: dorsal flexion of the wrist, 100°/100°; palmar flexion of the wrist, 95°/95°; pronation of the forearm, 90°/90°; and supination of the forearm, 90°/90° (Fig. 5c-f).

## Discussion and conclusions

Generally, open reduction of the DRUJ is required in a case of Galeazzi fracture-dislocation with interposition and an irreducible DRUJ. We searched for English literature published in PubMed, and we found that an irreducible DRUJ with Galeazzi fracture-dislocation in adults was reported in only 16 cases [1–9]. Patients’ mean age was 22 years (range, 16–40 years). Anatomical structures causing interposition of the DRUJ included the extensor carpi ulnaris (ECU) (10 cases), extensor digiti minimi (EDM) (3 cases), bone fragment of the styloid process (2 cases), capsule (1 case), extensor digitorum communis (EDC) (1 case), and soft tissue (2 cases). All cases were accompanied by an ulnar styloid fracture. The most common patterns of irreducibility were as follows: the extensor retinaculum and dorsal compartment were collapsed; bone fragment of the styloid process was translated volarly and the ulnar head was dislocated dorsally; and the ECU tendon, EDM, EDC, and extensor retinaculum were trapped in the DRUJ. To remove these interpositions, open reduction through a dorsal incision was required for all patients.

Kikuchi et al. reported a case of Galeazzi fracture-dislocation with an irreducible DRUJ due to entrapment of a fragment avulsed from the fovea of the ulna [4]. The fracture of the radius was treated with open reduction and internal fixation, but the ulnar head could not be reduced. Therefore, the ulnar head was exposed through a dorsal incision in order to reduce the DRUJ. Suspected mechanism of injury in our case was as follows: First, metaphyseal fracture of the radius occurred during a high-energy bicycle race. Second, after the radius was shortened, the dislocation of the DRUJ occurred, and it was accompanied by ulnar styloid fracture. The fragment of the ulnar styloid remained in its original position relative to the radius. Third, the doctor who treated it earlier could not manually reduce the ulnar styloid, but achieved reduction of the radius to some extent. Thus, the fragment of the ulnar styloid and TFCC were entrapped between the sigmoid notch and ulnar head.

Arthroscopy in the acute setting is applied for an assessment of displacement and reduction of an intra-articular fracture fragment and evaluation of the damage and repair of intra-articular ligament injury [11]. As was seen in our case, arthroscopy is also useful to assess the irreducible condition that inhibits reduction of the anatomical structure and to obtain reduction on direct vision. Dry technique might be a good option when the soft tissue damage is strong [12]. DRUJ arthroscopy is being adopted because of the recent developments in medical technology and improvement in surgical techniques. This technique has been recently used for arthroscopic repair for TFCC injury, resection of the ulnar head for ulnar abutment syndrome, and the arthroscopic Sauve-Kapandji procedure [13, 14]. When the incision for the dorsal capsule and ECU subsheath, which comprise the TFCC, was performed for open reduction, postoperative immobilization was necessary for several weeks. Moreover, an adhesion might occur through the open approach and induce the limited motion of the wrist and forearm. Although arthroscopic reduction is not indicated for all irreducible DRUJs, we consider that it is superior to the open surgical method in terms of evaluating interpositions and being minimally invasive. The technique we used enabled our patient to return early to professional sports activity.

In recent years, several studies have shown that the combined treatment of PTH and LIPUS accelerated fracture bone healing and enhanced bone properties in animal experiments and human clinical study [15, 16]. The authors of these studies concluded that combined therapy with PTH and LIPUS may become a useful option for treating acute fracture and elderly patients with lower limb fracture. Our patient had a strong desire to return to racing as early as possible because he wanted to take part in the championship tournament that was going to be held for rookies in another two months. Therefore, we proceeded aggressively with postoperative therapy, using arthroscopy without postoperative immobilization and early exercise for obtaining good range of motion, and PTH and LIPUS for achieving bone union earlier. This combined therapy might be a possible postoperative treatment for those who wish to achieve bone union earlier.

## Data Availability

Data are contained within the manuscript.

## Abbreviations

DRUJ: Distal radioulnar joint
ECU: Extensor carpi ulnaris
EDC: Extensor digitorum communis
EDM: Extensor digiti minimi
LIPUS: Low-intensity pulsed ultrasound
PTH: Parathyroid hormone
TBW: Tension band wiring
TFCC: Triangular fibrocartilage complex
